# Prognostic Value of CONUT and ALBI in Metastatic Pancreatic Cancer: A Retrospective Cohort Study

**DOI:** 10.3390/diagnostics15243161

**Published:** 2025-12-11

**Authors:** Pınar Peker, Aslı Geçgel, Serdar Yılmaz, Cafer Cırık, Seher Selvi, Berna Bozkurt Duman, Timuçin Çil

**Affiliations:** 1 Department of Medical Oncology, Adana City Training and Research Hospital, Adana 01370, Turkey; ssdrylmz@hotmail.com (S.Y.); doktorcafercirikhihi@gmail.com (C.C.); seherimselvim@gmail.com (S.S.); berboz@gmail.com (B.B.D.); drtimucincil@gmail.com (T.Ç.); 2 Department of Medical Oncology, Yozgat City Hospital, Yozgat 66000, Turkey; dr.asltrgt@gmail.com

**Keywords:** metastatic pancreatic cancer, CONUT score, ALBI grade, nutritional status, prognostic biomarkers, overall survival, inflammation-based indices

## Abstract

**Background:** Metastatic pancreatic cancer carries a poor prognosis, and reliable prognostic markers are needed to guide treatment decisions. The Controlling Nutritional Status (CONUT) score and Albumin-Bilirubin (ALBI) grade are simple measures of inflammatory, nutritional, and hepatic status, but their prognostic value in metastatic pancreatic cancer remains unclear. This study aimed to evaluate the prognostic significance of CONUT and ALBI scores in patients with metastatic pancreatic cancer. **Methods**: This retrospective study included 192 patients with metastatic pancreatic cancer treated at Adana City Education and Research Hospital between 1 May 2019, and 1 February 2025. Baseline CONUT scores and ALBI grades were calculated. All laboratory parameters, including those used for CONUT and ALBI calculation, were obtained prior to the initiation of any systemic therapy. Overall survival (OS) was analyzed using Kaplan–Meier survival curves and multivariate Cox proportional hazards regression to assess the independent prognostic value of both indices. **Results:** The median OS of the cohort was 14.0 months. Patients with high CONUT scores had significantly shorter OS compared to those with low–moderate scores (7.8 vs. 14.0 months, *p* < 0.001). Similarly, ALBI Grade 2 was associated with worse survival than ALBI Grade 1 (13.0 vs. 14.0 months, *p* = 0.049). In multivariate Cox regression analysis, high CONUT score (HR = 5.01, 95% CI: 2.27–10.98, *p* < 0.001) and ALBI Grade 2 (HR = 17.86, 95% CI: 9.63–31.04, *p* < 0.001) remained independent predictors of poor OS. **Conclusions:** The CONUT and ALBI scores are independent and clinically meaningful prognostic markers in metastatic pancreatic cancer. Their routine use may support risk-adapted, personalized treatment. These readily accessible biomarkers offer a simple and accessible tool to guide clinical decisions.

## 1. Introduction

Pancreatic adenocarcinoma is one of the deadliest malignancies, ranking among the leading causes of cancer-related mortality worldwide, with a 5-year survival rate of less than 10% despite recent therapeutic advances [1,2]. The metastatic stage is especially challenging, as treatment tolerance and outcomes vary widely among patients. Therefore, there is a need for simple and reliable prognostic markers to support risk stratification and guide clinical decision-making [3]. Systemic inflammation and nutritional status have recently gained attention as predictors of survival in several solid tumors. The Controlling Nutritional Status (CONUT) score (based on serum albumin, total cholesterol, and lymphocyte count) reflects both immune and nutritional reserve. A recent meta-analysis confirmed that elevated CONUT scores are associated with poorer overall survival in pancreatic cancer [4].

The Albumin–Bilirubin (ALBI) score, initially developed for assessing hepatic reserve in cirrhosis, also demonstrates prognostic value in several cancers, including pancreatic cancer with liver involvement [5]. Because CONUT and ALBI reflect different but clinically relevant dimensions of host physiology, they may assist in treatment selection and patient stratification.

Both CONUT and ALBI have been associated with prognosis in various malignancies. However, whether their combined use adds prognostic value in metastatic pancreatic cancer remains unclear. Notably, to our knowledge, there has been no report to date that has assessed both indices concurrently within a single metastatic population, compared their mutual contributions, and examined the pooled information from combining multiple factors for risk estimation beyond conventional clinical parameters. Since individuals with metastatic pancreatic cancer often have severe malnutrition, systemic inflammation, and impaired hepatic reserves, the combination of these two convenient scores can lead to a more complete predictive model. Thus, our study promises to uniquely address this gap by evaluating, for the first time, the independent and joint prognostic significance of CONUT and ALBI in a large real-world population of patients with metastatic pancreatic cancer.

Although both scores have demonstrated prognostic value individually across multiple malignancies, their combined predictive significance has not been examined in metastatic pancreatic cancer. Recent studies have integrated nutritional or inflammatory markers with liver function indices to improve survival prediction, such as combining myosteatosis with ALBI grade in hepatocellular carcinoma [6].

Despite growing evidence that CONUT and ALBI are independent prognostic indicators in several solid tumors, including pancreatic cancer, their combined prognostic value in the metastatic setting has not been investigated. Simultaneous evaluation of CONUT and ALBI may offer a more thorough and economical assessment of host physiological reserve and survival potential because patients with metastatic pancreatic cancer often present with both malnutrition and hepatic impairment due to tumor burden and systemic catabolic effects.

This study aimed to evaluate the prognostic value of CONUT and ALBI in metastatic pancreatic cancer and to determine their utility in clinical risk stratification.

## 2. Materials and Methods

### 2.1. Study Design and Patient Population

This single-center, retrospective cohort study was conducted at Adana City Training and Research Hospital (Adana, Türkiye) between 1 May 2019, and 1 February 2025. A total of 192 consecutive patients with histologically confirmed metastatic pancreatic adenocarcinoma were included. Eligibility criteria were (1) confirmed diagnosis of metastatic pancreatic cancer, (2) availability of baseline laboratory data required to calculate CONUT and ALBI scores, and (3) age ≥18 years. Patients were excluded if they had incomplete biomarker data, concurrent malignancies, or insufficient follow-up.

All laboratory values were obtained before initiation of systemic therapy. Because hepatic dysfunction in metastatic pancreatic cancer may arise from obstructive jaundice, cholestasis, systemic inflammation, or cachexia, ALBI was assessed in all patients irrespective of liver metastasis to reflect global hepatic reserve.

The CONUT score was used to evaluate immune–nutritional status and was calculated from serum albumin, total cholesterol, and lymphocyte count, with total scores categorized as low (0–1), moderate (2–4), or high (5–9) nutritional risk according to the original validated criteria [7].

The ALBI score was used to assess hepatic functional reserve and was calculated from serum albumin and total bilirubin, classifying patients into Grade 1 (≤−2.60) or Grade 2 (>−2.60) in accordance with established methodology [8]. Missing data were minimal (<5%) and limited to laboratory variables; these values were imputed using the cohort mean or median, depending on distribution, and no missing demographic or survival data were present. Scoring criteria for both indices are summarized in Table 1.

This study was approved by the Ethics Committee of Adana City Training and Research Hospital (Approval No: 748, Meeting No: 17, dated 25 September 2025) and was conducted in accordance with the principles of the Declaration of Helsinki. Due to the retrospective design of the study and the use of anonymized patient data, the requirement for informed consent was waived by the Ethics Committee.

### 2.2. Statistical Analysis

Statistical analyses were performed using IBM SPSS Statistics (Version 25.0, IBM Corp., Armonk, NY, USA). Continuous variables were summarized as mean ± standard deviation or median (IQR), and categorical variables as frequencies and percentages. Overall survival (OS) was defined as the time from metastatic diagnosis to death from any cause. OS was estimated using Kaplan–Meier curves, and differences between groups were compared using the log-rank test. We also constructed multivariate Cox proportional hazards models, which incorporated clinically important prognostic factors regardless of their univariate statistical significance (age, sex, ECOG PS, liver metastasis, and first-line regimens) as anchors to assess the independent effects of CONUT and ALBI. All statistical tests were two-sided, with significance set at *p* < 0.05. A sensitivity analysis was additionally performed to assess the robustness of the multivariate model. The proportional hazards assumption for the multivariate Cox model was evaluated using Schoenfeld residuals, and no significant violations were observed.

Given the modest sample size of this single-center cohort, the multivariate model may be prone to overfitting; therefore, effect estimates—particularly for ALBI Grade 2—should be interpreted with caution and validated in larger, multicenter populations.

## 3. Results

A total of 192 patients with metastatic pancreatic adenocarcinoma were included in the study. The median age was 64 years (range: 34–86), and 61.5% were male. Most patients had preserved performance status, with 91.1% classified as ECOG 0–1 and 8.9% as ECOG ≥2. CA 19–9 levels were ≤200 U/mL in 56.8% of patients and >200 U/mL in 43.2%. The mean LMR and NLR were 3.25 ± 2.06 and 4.50 ± 4.53, respectively, with 50.5% of patients having LMR <2.8. Liver metastasis was present in 40.1% of patients, lymph node metastasis in 88.0%, and bone metastasis in 13.5%. According to CONUT classification, 55.2% of patients were categorized as low risk, 40.6% as moderate risk, and 4.2% as high risk; based on ALBI grading, 46.9% were classified as Grade 1 and 53.1% as Grade 2. The median follow-up duration was 14.0 months (range: 5.0–21.0), and all patients had died by the end of follow-up. This value reflects the actual observed overall survival rather than median follow-up time, likely influenced by the high proportion of patients receiving combination chemotherapy, predominance of ECOG 0–1 status, and exclusion of patients with insufficient follow-up. Baseline characteristics are summarized in Table 2.

Age showed a minimal effect on overall survival (OS), with median OS of 14 months in patients ≤65 years and 13 months in those >65 years (*p* = 0.048). There was no significant difference in OS according to sex (*p* = 0.599), ECOG performance status (*p* = 0.175), or CA 19–9 levels (*p* = 0.988). LMR and NLR were also not associated with survival (*p* = 0.283 and *p* = 0.488, respectively). Similarly, the presence of liver metastasis (*p* = 0.608), lymph node metastasis (*p* = 0.812), or bone metastasis (*p* = 0.121) did not significantly affect OS. The distribution of CONUT categories in the cohort was as follows: low (55.2%), moderate (40.6%), and high (4.2%). This indicates that the highest-risk group comprised a small number of patients. In addition to such clinicopathological parameters, treatment-related variables are also an important part of the prognosis for patients. Thus, the distribution of first-line regimens, status of supportive care, and number of treatment lines in the cohort are shown (Table 3).

**Figure 1:**
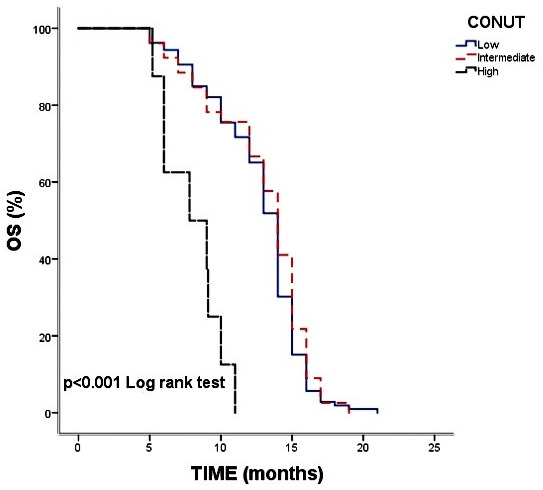
Kaplan–Meier Overall Survival Curves According to CONUT Score. Overall survival was significantly shorter in patients with high CONUT scores compared with those in the low–moderate risk groups (median OS: 7.8 vs. 14.0 months, *p* < 0.001).

**Figure 2:**
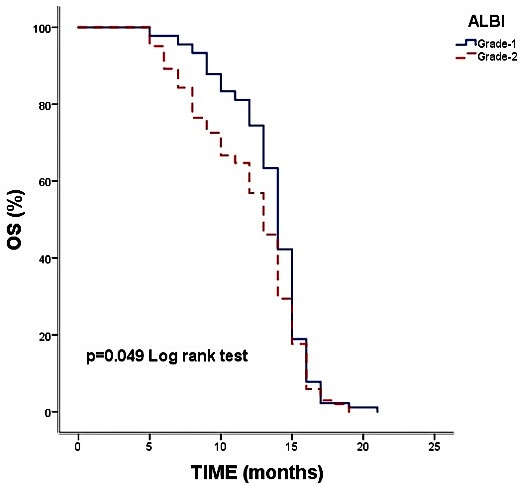
Kaplan–Meier Overall Survival Curves According to ALBI Grade. Overall survival was significantly shorter in patients with ALBI Grade 2 compared with those with ALBI Grade 1 (median OS: 13.0 vs. 14.0 months, *p* = 0.049).

In contrast, the CONUT score was strongly associated with prognosis. Patients with high CONUT scores had significantly shorter survival compared with those classified as low or moderate risk (median OS: 7.8 vs. 14.0 months, *p* < 0.001) (Figure 1). The ALBI score also demonstrated prognostic value, with median OS of 13 months in ALBI Grade 2 versus 14 months in ALBI Grade 1 (*p* = 0.049) (Figure 2). Overall survival across clinical subgroups is summarized in Table 4. Notably, the similarity in median OS across several subgroups reflects the narrow survival range of this uniformly advanced cohort rather than any rounding of raw data.

The multivariable Cox model included key clinical variables (age, sex, ECOG performance status, liver metastasis, and first-line treatment type) in addition to CONUT and ALBI, regardless of their univariate associations. Pancreatic cancer, particularly in its metastatic stage, remains one of the most lethal malignancies. In this clinically adjusted model, both CONUT and ALBI remained independent predictors of OS. Age did not remain statistically significant in the multivariate model (HR = 1.13, 95% CI: 0.84–1.53; *p* = 0.409). In contrast, a high CONUT score was independently associated with increased mortality (HR = 5.01, 95% CI: 2.27–10.98; *p* < 0.001). ALBI Grade 2 was also a strong independent predictor of worse survival (HR = 17.86, 95% CI: 9.63–31.04; *p* < 0.001). This prognostic effect persisted after adjustment for liver metastasis, supporting the clinical relevance of ALBI even in patients without hepatic involvement. The overall model demonstrated good fit (−2 log-likelihood *p* < 0.001). Detailed results are presented in Table 5.

## 4. Discussion

Pancreatic cancer, particularly in its metastatic stage, remains one of the most lethal malignancies, largely due to its aggressive biology, late diagnosis, and limited treatment responsiveness. Importantly, the independent associations of CONUT and ALBI persisted even after adjustment for major clinical confounders, including ECOG performance status, liver metastasis, and first-line treatment regimen.

The lack of a survival advantage for FOLFIRINOX in our cohort likely reflects real-world treatment limitations rather than an absence of biological efficacy. Many patients who initiated FOLFIRINOX were unable to complete the intended course due to toxicity, rapid clinical decline, or early progression, resulting in reduced dose intensity compared with clinical trial populations. Dose reductions and early discontinuations were common, and these factors were not fully captured in our retrospective dataset. Therefore, the expected OS benefit of FOLFIRINOX was likely attenuated, explaining the similarity in survival across first-line regimens.

Identifying reliable and easily accessible prognostic biomarkers is therefore essential to optimize treatment decisions and improve patient stratification. In this study, we evaluated the prognostic significance of the CONUT and ALBI scores in metastatic pancreatic cancer and found that both indices served as independent predictors of overall survival. These findings underscore the importance of nutritional, inflammatory, and hepatic functional status in shaping clinical outcomes.

The CONUT score, which incorporates serum albumin, total cholesterol, and lymphocyte count to capture immune–nutritional status, showed a strong association with survival. Patients with high CONUT scores had significantly worse outcomes, reflecting the negative prognostic impact of malnutrition and systemic inflammation. Because only eight patients fell into the high CONUT category, the precision of effect estimates in this subgroup is limited. The survival curves of the low and moderate CONUT groups were nearly overlapping, indicating that the overall prognostic signal was primarily driven by the eight high-risk patients with markedly poor outcomes. This distributional imbalance limits the robustness of subgroup comparisons, and therefore, these findings should be interpreted with caution and considered hypothesis-generating. The direction of the association is consistent with prior evidence, but these findings should be interpreted cautiously. Our results are consistent with prior evidence, including a meta-analysis by Niu and Yan (2023), which reported that elevated CONUT scores were significantly associated with poorer overall survival in gynecologic malignancies (HR = 1.52; 95% CI: 1.13–2.04) [9]. Similar associations have also been demonstrated in colorectal cancer, where high CONUT scores predicted increased mortality and poorer treatment tolerance [10,11].

Furthermore, the ALBI score was significantly associated with survival in our cohort. Although ALBI was originally validated in cirrhosis and liver-dominant malignancies, hepatic dysfunction in metastatic pancreatic cancer frequently arises even without liver metastasis due to obstructive jaundice, cholestasis, systemic inflammation, cancer-associated cachexia, and subclinical hepatic impairment. These mechanisms alter albumin and bilirubin independently of metastatic pattern, providing a physiological basis for the use of ALBI in the entire metastatic cohort. Nevertheless, we acknowledge that subgroup imbalances, particularly the small high-risk cohorts, limit statistical precision, and these findings should be interpreted cautiously and considered hypothesis-generating.

Patients classified as ALBI Grade 2 demonstrated shorter overall survival compared with those in Grade 1. Although the association was statistically significant, the effect size observed in the multivariate model (HR = 17.86) was notably large and should be interpreted with caution. Given the small size of the ALBI Grade 2 subgroup, this estimate may reflect statistical inflation. Although ALBI Grade 2 included 102 patients, the apparent ‘small subgroup’ effect reflects distributional sparsity after adjustment for multiple covariates rather than absolute sample size. Such imbalance can inflate hazard ratio estimates despite a moderate group size. This may be partially influenced by subgroup size, overlap between hepatic function and systemic inflammation markers, and the inherent limitations of single-center datasets. While our collinearity diagnostics did not indicate substantial redundancy, external validation is necessary to confirm the magnitude of this effect. We also performed a sensitivity analysis, and although ALBI Grade 2 remained statistically significant, the effect size was attenuated, suggesting that the originally high HR was partly influenced by subgroup size and model instability.

These findings are consistent with previous studies in hepatocellular carcinoma, where ALBI has been validated as a reliable, objective marker of hepatic reserve and prognosis [12]. Similar evidence also supports the prognostic value of ALBI in pancreatic cancer, particularly in patients with liver involvement, reinforcing its relevance in this clinical context [5,13,14]. Recent studies published in 2024–2025 have similarly demonstrated that inflammatory-nutritional indices, including SII, PNI, and CRP-based markers, retain independent prognostic value in metastatic pancreatic cancer, further supporting the relevance of such composite scores in advanced disease [15,16,17].

Taken together, our findings indicate that both CONUT and ALBI are useful, practical prognostic indicators in metastatic pancreatic cancer. In the context of existing prognostic systems, CONUT and ALBI complement established inflammation-nutritional markers such as the Glasgow Prognostic Score (GPS), Prognostic Nutritional Index (PNI), and the modified Systemic Inflammation Index (mSII). GPS and mSII primarily capture systemic inflammation, while PNI reflects immunonutritional status; however, these tools do not directly incorporate hepatic functional reserve. In contrast, the ALBI score objectively quantifies liver function without relying on subjective assessments, and CONUT additionally integrates lipid metabolism and lymphocyte-mediated immunity. Recent studies have shown that PNI, SII/mSII, and CRP-based indices retain prognostic value in metastatic pancreatic cancer, but their predictive strength may vary across heterogeneous clinical settings. Our results suggest that CONUT and ALBI provide complementary information, particularly in patients with coexisting malnutrition and impaired hepatic reserve, and may enhance risk stratification when used alongside or in place of traditional indices.

However, validation in larger, multi-center cohorts is needed before these markers can be widely implemented in clinical practice. From a clinical point of view, both CONUT and ALBI may be readily applicable during the clinical courses, since they can be estimated using routine parameters measured at the time of diagnosis. CONUT may be able to predict patients who may require early nutritional improvement, dietitian consultation, or supportive measures prior to systemic treatment. ALBI offers an objective tool to evaluate hepatic functional reserve, which could be used for the choice of chemotherapy intensity, prediction of toxicity, and dose modifications. In a disease rife with malnutrition, systemic inflammation, and liver dysfunction, such as metastatic pancreatic cancer, the application of CONUT and ALBI scores together may allow for risk-adapted treatment selection, closer patient monitoring, and more personalized decision-making without additional cost or procedure burden.

To our knowledge, this is the first study to assess both CONUT and ALBI scores in metastatic pancreatic cancer, comparing their prognostic impact within a single cohort. Previous research evaluated these scores individually in localized or surgical settings, without addressing their combined predictive value in metastatic disease, where nutritional depletion and hepatic dysfunction are more prominent. Our findings show that both parameters retain independent prognostic value and offer additive information when analyzed together. Therefore, these cut-offs could enable a more powerful and practical estimation approach in daily oncology practice.

In our study, conventional prognostic factors such as age, sex, and the presence of liver metastasis did not demonstrate a significant association with overall survival. Notably, the commonly used tumor marker CA 19–9 also showed no prognostic relevance in this cohort, which aligns with the findings of Mirkin et al., who reported substantial variability in CA 19–9 levels across patients and limited prognostic utility in advanced disease [18]. While CA 19–9 may reflect tumor burden, its value in predicting survival appears limited in metastatic settings. In contrast, the CONUT and ALBI scores provided more consistent prognostic information, suggesting that immune-nutritional and hepatic functional status may serve as more reliable indicators of survival in metastatic pancreatic cancer [19].

It is important to use caution when interpreting the lack of a strong correlation between our patients’ survival, liver metastases, and CA 19–9. However, it is more likely that these null results are the result of sparse variation between cases rather than a true lack of prognostic significance, given the uniformly late stage of disease in this study, a narrow range of survival, and a relatively small sample size for a single-center analysis. Therefore, our results do not contradict the data that is already available; rather, they only show how challenging it is to distinguish an OS difference across individuals with similar advanced metastatic stages.

Other clinical variables such as sex, liver metastasis, and lymph node involvement did not significantly influence survival in our cohort, despite liver metastasis being traditionally associated with poor outcomes in pancreatic cancer. In contrast, systemic inflammation and nutritional status (captured by the CONUT and ALBI scores) demonstrated a stronger prognostic impact and may refine conventional risk stratification models [20]. The association between high CONUT scores and poor survival is consistent with patterns observed in other malignancies in which malnutrition and inflammation contribute to disease progression and reduced treatment tolerance [21].

The CONUT score can help identify patients who may benefit from early nutritional optimization and supportive interventions, while the ALBI score provides an objective assessment of hepatic functional reserve to guide treatment decisions and monitoring [22,23]. Both scores are inexpensive, require only routine laboratory parameters, and are simple to calculate, making them practical prognostic tools, particularly in resource-limited settings.

Despite the strengths of this study, further validation is required. Because our analysis was based on a single-center cohort, the prognostic utility of CONUT and ALBI should be confirmed in larger, multi-center prospective studies. Future research should also assess whether interventions targeting malnutrition and systemic inflammation could improve outcomes in patients with high CONUT scores [24] and whether integrating these indices with other emerging biomarkers may enhance personalized treatment strategies in metastatic pancreatic cancer [25].

This study has several limitations. It was based on a single-center retrospective cohort, which may limit the generalizability of the findings. In addition, the relatively small number of patients in the ALBI Grade 2 subgroup may have contributed to the notably high hazard ratio observed for this category, suggesting the possibility of statistical inflation rather than a biological effect of this magnitude. Although multicollinearity diagnostics did not indicate substantial overlap between variables, validation in larger, multi-center cohorts is needed to confirm the robustness of these results.

In conclusion, our findings indicate that the CONUT and ALBI scores are reliable and independent prognostic markers in metastatic pancreatic cancer. These scores reflect systemic inflammation, nutritional status, and hepatic functional reserve, factors that play crucial roles in patient survival. Incorporating CONUT and ALBI into routine clinical assessment may improve patient stratification and support more personalized treatment decision-making. Future prospective, multi-institutional studies are needed to validate these results and determine whether integrating CONUT and ALBI with emerging molecular or immunologic markers can enhance individualized treatment strategies in metastatic pancreatic cancer.

## Figures and Tables

**Table 1:** Calculation and Risk Classification of CONUT and ALBI Scores.

Score System	Component/Formula	Value Range	Score/Category
CONUT Score	Serum Albumin (g/dL)	≥3.5	0
		3.0–3.4	1
		2.5–2.9	2
		<2.5	3
	Total Cholesterol (mg/dL)	≥180	0
		140–179	1
		100–139	2
		<100	3
	Lymphocyte Count (cells/μL)	≥1600	0
		1200–1599	1
		800–1199	2
		<800	3
	Total CONUT Score	0–9	—
	Risk Classification	0–1	Low risk
		2–4	Moderate risk
		5–9	High risk
ALBI Score	Formula	—	ALBI = (0.66 × log10(bilirubin μmol/L)) − (0.085 × albumin g/L)
	Risk Classification	≤−2.60	Grade 1 (well-preserved liver function)
		>−2.60	Grade 2 (impaired liver function)

**Table 2:** Baseline Sociodemographic and Clinical Characteristics of the Study Cohort. This table summarizes the demographic features, performance status, disease distribution, tumor markers, inflammatory indices, and nutritional and hepatic function parameters (CONUT and ALBI scores) of the patients included in the study.

Variable	Value
**Age (mean ± SD)**	63.15 ± 9.39 years
**Age (median, range)**	64 (34–86)
**Age ≤65 years (%)**	59.4%
**Age >65 years (%)**	40.6%
**Gender (Male/Female) (%)**	61.5%/38.5%
**ECOG Performance Status (0–1/≥2) (%)**	91.1% 8.9%
**Pre-chemotherapy CA 19–9 ≤ 200 (>200) (%)**	56.8% (≤200)/43.2% (>200)
**LMR (mean ± SD, median, range)**	3.25 ± 2.06, 2.8 (0.30–13.00)
**LMR <2.8/>2.8 (%)**	50.5%/49.5%
**NLR (mean ± SD, median, range)**	4.50 ± 4.53, 3.0 (0.70–32.50)
**NLR <3/>3 (%)**	51.0%/49.0%
**Liver Metastasis (Yes/No) (%)**	40.1%/59.9%
**Lymph Node Metastasis (Yes/No) (%)**	88.0%/12.0%
**Bone Metastasis (Yes/No) (%)**	13.5%/86.5%
**CONUT Score (Low/Moderate/High)**	55.2%/40.6%/4.2%
**ALBI Score (Grade 1/Grade 2)**	46.9%/53.1%
**Follow-up Duration (mean ± SD, median, range)**	13.24 ± 3.17 months, 14.0 (5.0–21.0 months)

**Table 3:** Distribution of treatment patterns in the study cohort. This table outlines the first-line regimens, supportive care use, and treatment-line distribution in patients with metastatic pancreatic cancer.

Treatment Category	Number of Patients	Percentage (%)	Notes
FOLFIRINOX	78	40.6	First-line regimen
Gemcitabine + Nab-paclitaxel	18	9.4	First-line regimen
Gemcitabine monotherapy	82	42.7	Used in frail/ECOG ≥ 2
Best supportive care only	14	7.3	No systemic therapy
≥2 lines of therapy	64	33.3	Received second-line or more

**Table 4:** Comparison of Overall Survival Across Clinical Subgroups. This table summarizes overall survival (OS) according to age, sex, performance status, metastatic distribution, and CONUT and ALBI classifications. OS was estimated using the Kaplan–Meier method, and comparisons were made using the log-rank test. Differences with *p* < 0.05 were considered statistically significant.

Variable	1-Year OS (%)	Median OS (Months, 95% CI)	*p*-Value
**General**	78.1%	14.00 (13.62–14.37)	N/A
**Age**			
≤65 years	74.6%	14.00 (13.49–14.50)	0.048
>65 years	56.4%	13.00 (12.28–13.71)	N/A
**Gender**			
Male	74.6%	14.00 (13.41–14.58)	0.599
Female	77.0%	14.00 (13.54–14.45)	
**ECOG Performance Status**			
0–1	77.1%	14.00 (13.61–14.38)	0.175
≥2	88.2%	14.00 (11.98–16.01)	
**Pre-chemotherapy CA 19–9**			
≤200	69.7%	14.00 (13.47–14.53)	0.988
>200	72.3%	14.00 (13.48–14.51)	
**LMR**			
<2.8	78.4%	14.00 (13.40–14.59)	0.283
>2.8	77.9%	14.00 (13.53–14.46)	
**NLR**			
<3	78.6%	14.00 (13.50–14.50)	0.488
>3	77.7%	14.00 (13.44–14.55)	
**Liver Metastasis**			
Yes	79.1%	14.00 (13.52–14.47)	0.608
No	76.6%	14.00 (13.33–14.66)	
**Lymph Node Metastasis**			
Yes	56.5%	14.00 (11.70–16.29)	0.812
No	79.3%	14.00 (13.62–14.38)	
**Bone Metastasis**			
Yes	79.5%	14.00 (13.61–14.39)	0.121
No	69.2%	13.00 (11.76–14.23)	
**CONUT Score**			
Low	78.3%	14.00 (13.57–14.42)	<0.001
Moderate	78.2%	14.00 (13.34–14.65)	
High	12.5%	7.80 (3.64–11.95)	
**ALBI Score**			
Grade 1	74.4%	14.00 (13.51–14.48)	0.049
Grade 2	56.9%	13.00 (12.29–13.70)	

**Table 5:** Multivariate Cox Regression Analysis of Clinical Variables Associated with Mortality Risk. This table summarizes the independent effects of age, CONUT score, Ecog, Liver metastasis, First-line regimen and ALBI grade on mortality in patients with metastatic pancreatic cancer. The overall model demonstrated good fit (–2 Log Likelihood = 1684.11, *p* < 0.001).

Variables	HR (95% CI)	*p*
**Age**		
≤65	Ref (Reference)	
>65	1.13 (0.84–1.53)	0.409
**CONUT**		
Low	Ref (Reference)	
Moderate	0.85 (0.63–1.15)	0.315
High	5.01 (2.27–10.98)	<0.001
**ALBI**		
Grade 1	Ref (Reference)	
Grade 2	17.86 (9.63–31.04)	<0.001
**ECOG**		
0–1	Ref(Reference)	
>2	1.41 (0.92–2.15)	0.112
**Liver Metastasis**		
No	Ref(Reference)	
Yes	1.18 (0.79–1.76)	0.416
**First-line regimen**		
Folfirox	Ref(Reference)	
Others	0.74 (0.51–1.09)	0.127

## Data Availability

The original contributions presented in this study are included in the article. Further inquiries can be directed to the corresponding author.

## Abbreviations

**CONUT**	Controlling Nutritional Status
**ALBI**	Albumin–Bilirubin
**LMR**	Lymphocyte-to-Monocyte Ratio
**NLR**	Neutrophil-to-Lymphocyte Ratio
**ECOG**	Eastern Cooperative Oncology Group
**OS**	Overall Survival
**HR**	Hazard Ratio
**CI**	Confidence Interval
